# Molecular and Regulatory Mechanisms of Desensitization and Resensitization of GABA_A_ Receptors with a Special Reference to Propofol/Barbiturate

**DOI:** 10.3390/ijms21020563

**Published:** 2020-01-15

**Authors:** Youngnam Kang, Mitsuru Saito, Hiroki Toyoda

**Affiliations:** 1 Department of Behavioral Physiology, Graduate School of Human Sciences, Osaka University, Osaka 565-0871, Japan; 2 Department of Neurobiology and Physiology, School of Dentistry, Seoul National University, Seoul 110-749, Korea; 3 Department of Oral Physiology, Graduate School of Medical and Dental Sciences, Kagoshima University, Sakuragaoka 8-35-1, Kagoshima 890-8544, Japan; mtrsaito@dent.kagoshima-u.ac.jp; 4 Department of Neuroscience and Oral Physiology, Osaka University Graduate School of Dentistry, Osaka 565-0871, Japan

**Keywords:** GABA_A_ receptor, desensitization, resensitization

## Abstract

It is known that desensitization of GABA_A_ receptor (GABA_A_R)-mediated currents is paradoxically correlated with the slowdown of their deactivation, i.e., resensitization. It has been shown that an upregulation of calcineurin enhances the desensitization of GABA_A_R-mediated currents but paradoxically prolongs the decay phase of inhibitory postsynaptic currents/potentials without appreciable diminution of their amplitudes. The paradoxical correlation between desensitization and resensitization of GABA_A_R-mediated currents can be more clearly seen in response to a prolonged application of GABA to allow more desensitization, instead of brief pulse used in previous studies. Indeed, hump-like GABA_A_R currents were produced after a strong desensitization at the offset of a prolonged puff application of GABA in pyramidal cells of the barrel cortex, in which calcineurin activity was enhanced by deleting phospholipase C-related catalytically inactive proteins to enhance the desensitization/resensitization of GABA_A_R-mediated currents. Hump-like GABA_A_R currents were also evoked at the offset of propofol or barbiturate applications in hippocampal or sensory neurons, but not GABA applications. Propofol and barbiturate are useful to treat benzodiazepine/alcohol withdrawal syndrome, suggesting that regulatory mechanisms of desensitization/resensitization of GABA_A_R-mediated currents are important in understanding benzodiazepine/alcohol withdrawal syndrome. In this review, we will discuss the molecular and regulatory mechanisms underlying the desensitization and resensitization of GABA_A_R-mediated currents and their functional significances.

## 1. Introduction

Ligand-gated channels open in response to the neurotransmitter binding but also close (desensitize) for long periods with the agonist still bound [1,2]. It is demonstrated that desensitization of GABA_A_ receptor (GABA_A_R)-mediated currents is paradoxically correlated with the slowdown of their deactivation, i.e., resensitization [3]. Desensitization tends to prolong inhibitory currents and keeps the transmitter in the bound state of GABA_A_Rs. The rate at which the receptors enter the desensitization state will affect the shape of inhibitory currents [4,5,6].

The desensitization of GABA_A_R-mediated currents is modulated by various signal transductions. The PKA-mediated phosphorylation modulates the desensitization of GABA_A_R-mediated currents in chick cortical neurons [7], rat sympathetic ganglion neurons [8], rat cerebellar granule neurons [9], and recombinant GABA_A_Rs [10]. The PKC- and PKG-mediated phosphorylation decreases the fast component of desensitization in recombinant α1β1 GABA_A_Rs [11] and rat cerebellar granule cells [9], respectively. CaMKII (Ca^2+^/calmodulin-dependent protein kinase II) decreased the desensitization of GABA_A_R-mediated currents in rat spinal dorsal horn neurons [12], while calcineurin enhanced the desensitization of GABA_A_R-mediated currents in rat hippocampal neurons [13]. Calcineurin directly binds to the intracellular loop of the GABA_A_R γ2 subunit, thereby dephosphorylating the receptor [14]. Interestingly, it is reported that the desensitization of GABA_A_R-mediated currents, which is caused by the enhanced calcineurin activity, paradoxically prolongs the decay phase of inhibitory postsynaptic currents/potentials without appreciable diminution of their amplitudes [4].

The paradoxical correlation between desensitization and resensitization of GABA_A_R-mediated currents can be seen in response to a brief pulse in previous studies [3,4]. However, this relationship can be more clearly seen in response to a prolonged application of GABA for enough time to allow full desensitization. Indeed, hump-like GABA_A_R currents were produced after a strong desensitization at the offset of puff applications of GABA for 2 s in pyramidal cells of the barrel cortex in the phospholipase C-related catalytically inactive proteins (PRIP-1/2) double-knockout (PRIP-DKO) mice [15]. In these neurons, the increased calcineurin activity due to the potentiated Ca^2+^-induced Ca^2+^ release (CICR) and store-operated Ca^2+^ entry (SOCE) enhances the desensitization of GABA_A_R-mediated currents and subsequently causes resensitization of GABA_A_R-mediated currents [15]. GABARAP (GABA_A_R-associated protein) plays an important role in intracellular trafficking/clustering of GABA_A_Rs [16,17] and the clustered GABA_A_Rs display lower apparent affinity for GABA, faster deactivation, and slower desensitization [18]. The kinases and molecules involved in desensitization and resensitization (slowdown of deactivation) of GABA_A_R-mediated currents are summarized in Table 1.

Hump-like GABA_A_R currents after a strong desensitization were also seen at the offset of propofol applications at a high concentration (600 μM) in hippocampal pyramidal neurons [19], etomidate applications at a high concentration (1 mM) in rat spinal dorsal horn neurons [20], pentobarbital applications at high concentrations (1–3 mM) in frog sensory neurons [21,22], rat hippocampal neurons [23], and recombinant GABA_A_Rs [24,25,26,27,28,29] or phenobarbital applications at a high concentration (10 mM) in rat hippocampal neurons [23], although these were not seen at the offset of GABA applications. Drugs that cause desensitization and resensitization of GABA_A_R-mediated currents are summarized in Table 2. It is believed that the generation of hump-like currents may be caused by the removal of the blockade by anesthetic agents as partial antagonists [24], although their mechanisms remain unclear and the involvement of desensitization is not necessarily denied. Propofol and barbiturate are clinically used for treatment of benzodiazepine/alcohol withdrawal syndrome [30,31,32]. Considering that hump-like GABA_A_R currents that are seen after a strong desensitization or blockade were evoked at the offset of propofol or barbiturate applications, the regulatory mechanisms of desensitization/resensitization of GABA_A_R-mediated currents might be important for understanding benzodiazepine/alcohol withdrawal syndrome. Here, we discuss the molecular and regulatory mechanisms underlying the desensitization and resensitization of GABA_A_R-mediated currents in neurons of PRIP-DKO mice and their functional significances.

## 2. PRIP-1/2 are Involved in Desensitization and Resensitization of GABA_A_R-Mediated Currents

**Figure 1:**
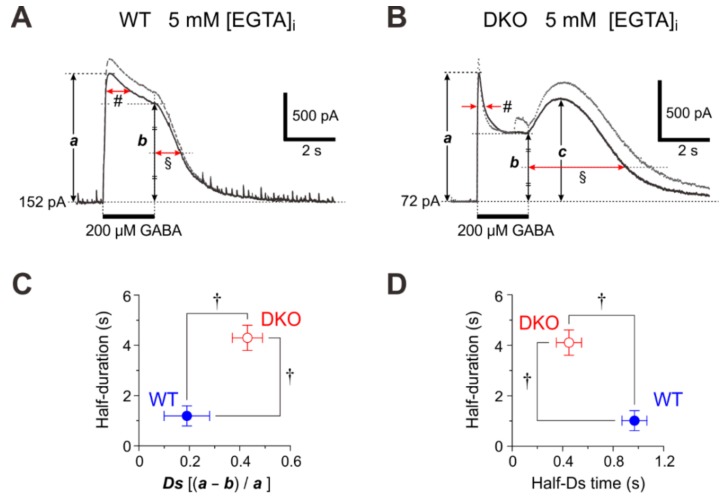
GABA_A_R-mediated currents evoked by GABA puff applications in wild-type and PRIP-DKO pyramidal cells. (**A** and **B**) Sample traces of GABA_A_R-mediated currents evoked at 0 mV in wild-type and PRIP-DKO pyramidal cells dialyzed with 5 mM EGTA, respectively, by puff application (4 and 6 psi) of GABA for 2 s. ***a***, ***b***, and ***c*** are the peak amplitude, the amplitude at the offset of the puff application, and the peak amplitude after the offset of the puff application, respectively. # and § are the durations at half amplitudes of desensitized component ([(a + b)/2]) and of tail-currents, respectively. (**C**) The relationship between the desensitization degree [Ds = (a – b)/a] of the GABA_A_R-mediated currents and half-duration of the tail-current (§) induced by a puff with 4 psi. †: *p* <0.01. (D) The relationship between the half-desensitization time of the GABA_A_R-mediated currents (#) and half-duration of the tail-current (§) induced by a puff with 4 psi. †: *p* <0.01. Adopted from [15].

PRIP-1/2 are involved in the membrane trafficking of GABA_A_Rs and the regulation of intracellular Ca^2+^ stores [16,17]. Thus, it was investigated whether and how the deletion of PRIP-1/2 affects GABA_A_R-mediated currents evoked by puff applications of GABA in layer III pyramidal cells of the barrel cortex. It was found that the deletion of PRIP-1/2 enhanced the desensitization of GABA_A_R-mediated currents but paradoxically induced a hump-like tail-current at the offset of the GABA puff (Figure 1) [15]. Thus, it is likely that PRIP-1/2 are involved in the desensitization and resensitization of GABA_A_R-mediated currents. Although similar tail-currents were observed following the removal of propofol [19], etomidate [20], pentobarbital [21,22,23,24,25,26,27,28,29], and phenobarbital [23], it was the first report on such hump-like tail-currents that were induced by GABA itself.

## 3. [Ca^2+^]_i_ Dependence of Desensitization and Resensitization of GABA_A_R-Mediated Currents and Their Abolishment by a Calcineurin Inhibitor

It is well known that the desensitization of GABA_A_R-mediated currents is accelerated by increases in [Ca^2+^]_i_ [33,34]. As expected, it was clearly demonstrated that both the acceleration of desensitization of GABA_A_R-mediated currents and the generation of the hump-like tail-currents were caused by increases in [Ca^2+^]_i_ [15]. Consistent with the idea that desensitization is mechanistically related to the deactivation of GABA_A_R-mediated currents [3], the progress of desensitization of GABA_A_R-mediated currents was invariably accompanied by the enhancement of the hump-like tail-currents [15]. These results suggested that the deletion of PRIP-1/2 results in an enhancement of the desensitization and resensitization of GABA_A_R-mediated currents through increases in [Ca^2+^]_i_. The involvement of CICR and the following SOCE in both the desensitization of GABA_A_R-mediated currents and the generation of the hump-like tail-currents in PRIP-DKO pyramidal cells was also demonstrated by an intracellular application of ruthenium red [15].

It has been demonstrated that a calcineurin inhibitor, cyclosporin A-cyclophilin A complex, suppressed the desensitization of GABA_A_R-mediated currents in acutely dissociated hippocampal neurons [13]. It has also been reported that the inhibition of calcineurin increased the rate of GABA unbinding from GABA_A_Rs [4]. Consistent with these previous studies, the bath application of a calcineurin inhibitor, fenvalerate, alleviated the desensitization of GABA_A_R-mediated currents and markedly decreased the hump-like tail-currents [15]. Thus, it is likely that the hump-like tail-currents in PRIP-DKO pyramidal cells were generated as a result of an acceleration of desensitization of GABA_A_R-mediated currents coupled with a slowdown of the GABA unbinding, which was mediated by Ca^2+^-dependent activation of calcineurin. Furthermore, Ca^2+^ imaging revealed that CICR and the following SOCE were more potent in PRIP-DKO pyramidal cells than in wild-type pyramidal cells [15]. Taken together, these results strongly suggest that the enhancement of desensitization and resensitization of GABA_A_R-mediated currents in PRIP-DKO pyramidal cells was largely mediated by the upregulation of Ca^2+^-dependent activity of calcineurin due to the potentiation of CICR followed by SOCE.

## 4. Deletion of PRIP-1/2 Prolongs eIPSCs in Layer II/III Pyramidal Cells

The differences in the kinetic properties of GABA_A_R-mediated currents between pyramidal cells of wild-type and PRIP-DKO mice should be reflected in the difference in inhibitory postsynaptic responses. Then, it was investigated how inhibitory postsynaptic responses reflect the changes in the kinetic properties of the GABA_A_R-mediated currents in layer III pyramidal cells of the PRIP-DKO barrel cortex.

**Figure 2:**
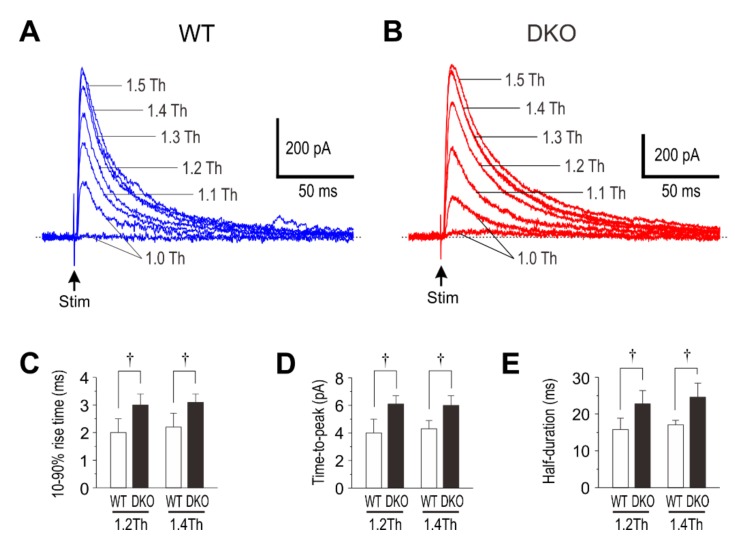
Evoked IPSCs (eIPSCs) in wild-type and PRIP-DKO pyramidal cells. (**A** and **B**) Superimposed sample traces of IPSCs evoked by stimulation with 1.0–1.5 times threshold (1.0–1.5 Th) in wild-type (**A**) and PRIP-DKO pyramidal cells (**B**). (**C**) The mean 10%–90% rise times of IPSCs evoked by stimulation with 1.2 Th in wild-type (*n* = 8) and PRIP-DKO pyramidal cells (*n* = 7) and those evoked by stimulation with 1.4 Th in wild-type (*n* = 8) and PRIP-DKO pyramidal cells (*n* = 7). †: *p* <0.01. (**D**) The mean times-to-peak of IPSCs evoked by stimulation with 1.2 Th in wild-type (*n* = 8) and PRIP-DKO pyramidal cells (*n* = 7) and those evoked by stimulation with 1.4 Th in wild-type (*n* = 8) and PRIP-DKO pyramidal cells (*n* = 7). †: *p* <0.01. (**E**) The mean half-durations of IPSCs evoked by stimulation with 1.2 Th in wild-type (*n* = 8) and PRIP-DKO pyramidal cells (*n* = 7) and those evoked by stimulation with 1.4 Th in wild-type (*n* = 8) and PRIP-DKO pyramidal cells (*n* = 7). †: *p* <0.01. Adopted from [35].

It was found that the deletion of PRIP-1/2 resulted in the prolongation of the decay phase of inhibitory postsynaptic currents/potentials (IPSCs/IPSPs) in layer II/III pyramidal cells evoked by stimulation of layer III (Figure 2), leaving the overall features of miniature IPSCs unchanged [35]. These observations suggest that the prolongation of inhibitory synaptic actions is likely to result from an enhancement of desensitization followed by an enhanced resensitization of GABA_A_R-mediated currents. It has been reported that the PRIP-DKO mice exhibited a reduced expression of synaptic GABA_A_Rs containing γ2 subunits by 40% in hippocampal neurons [36] and by 18% in cerebellar granule cells [37] as a consequence of the lack of binding between PRIP-1/2 and GABA_A_R-associated protein [38]. The mean peak amplitudes of the IPSCs and IPSPs in the PRIP-DKO pyramidal cells were not significantly different from those in the wild-type pyramidal cells. In any case, the amplitude of eIPSPs would not be increased by deletion of PRIP-1/2 [35]. Then, an increase in duration instead of amplitude of eIPSPs is likely to be caused in PRIP-DKO mice.

## 5. A Possible Kinetic Mechanism Underlying the Generation of the Hump-Like Tail-Currents and the Prolongation of eIPSCs

**Figure 3:**
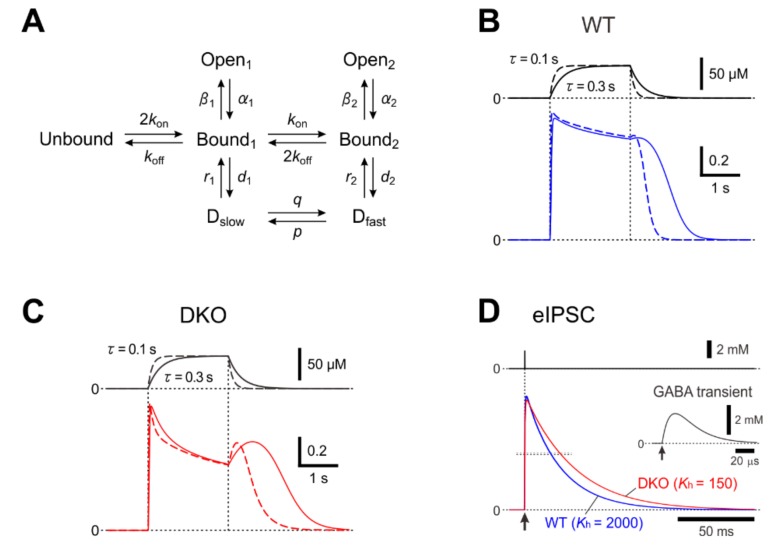
A kinetic model for a hump-like tail-current. (**A**) A kinetic model of GABA_A_Rs representing mono- and double-liganded states, each providing access to open and desensitized states. (**B** and **C**) Top; Presumed [GABA] changes created by puff application of GABA with a rectangular pressure pulse through a puff pipette containing 200 μM GABA in the extracellular medium was assumed to be diluted 4 times and the onset and offset of the puff application were assumed to be attenuated with a time constant ranging between 0.1 and 0.3 s. Bottom; superimposed traces of the simulated GABA_A_R-mediated currents under the condition that the attenuation time constant is 0.3 and 0.1 s (solid and interrupted traces, respectively) in simulated wild-type (**B**) and PRIP-DKO (**C**) pyramidal cells. The rate constants were as follows (in s^−1^): *k*_on_ = 15 μM^−1^, *β*_2_ = 2500, *α*_2_ = 142, *r*_2_ = 50, *β*_1_ = 200, *α*_1_ = 1100_,_ *r*_1_ = 0.35, *d*_1_ = 6, *q* = 1 × 10^−8^ μM^−1^, and *p* = 1. The values of *k*_off_ in WT and PRIP-DKO GABA_A_Rs were 90 and 30 s^−1^, respectively. The value of *d*_max_ in WT and PRIP-DKO GABA_A_Rs was 3600. The values of *k*_h_ in WT and PRIP-DKO GABA_A_Rs were 2000 and 200, respectively. (**D**) Superimposed traces of a simulated wild-type and PRIP-DKO eIPSC induced by a GABA transient shown on an expanded time scale (inset) with a small maximum conductance. The rate constants were as follows (in s^−1^): *k*_on_ = 20 μM^−1^, *β*_2_ = 2500, *α*_2_ = 195, *r*_2_ = 55, *β*_1_ = 100, *α*_1_ = 600_,_ *r*_1_ = 0.35, *d*_1_ = 11, *q* = 1 × 10^−8^ μM^−1^, *p* = 0, and *d*_max_ = 3100. The values of *k*_off_ in WT and PRIP-DKO GABA_A_Rs were 550 and 410 s^−1^, respectively. The value of *d*_max_ in WT and PRIP-DKO GABA_A_Rs was 310. The values of *k*_h_ in WT and PRIP-DKO GABA_A_Rs were 2000 and 150, respectively. Adopted from [15] and [35].

To understand the kinetic mechanisms underlying the generation of the hump-like tail-currents and the prolongation of eIPSCs, these currents were simulated using a previously proposed model [3] (Figure 3). It was examined whether the possible increase in the fast desensitization rate (*d*_2_) and the possible decrease in the unbinding rate (*k*_off_) can lead to a generation of the hump-like tail-current at the offset of the GABA puff.

It is known that GABA binding affinity was much larger in the desensitized GABA_A_Rs compared to the non-desensitized GABA_A_Rs and the binding affinity of the desensitized GABA_A_Rs increased depending on the concentration of the pre-applied GABA as was the case with the degree of desensitization of GABA_A_R-mediated currents [39]. Then, when the probability of being in the desensitized state (D_fast_) for GABA_A_Rs was increased by increasing GABA concentration ([GABA]) or during the 2 s puff application of GABA, D_fast_ would be further recruited, leaving Open_2_ unchanged. Thus, it is reasonable to assume that the *d*_2_, but not *β*_2_, increase in a manner dependent on [GABA] [15,39]. Because Bound_2_, which is bifurcated into Open_2_ and D_fast_, increases in a manner dependent on [GABA], the idea was incorporated in this model by defining *d*_2_ as follows;

$$d_{2}=\frac{\;d_{max}}{1+\;{\left(\frac{K_{h}}{\left[\mathrm{GABA}\right]}\right)}^{n}}$$

where *d*_max_ is the maximum desensitization rate, *K*_h_ is the [GABA] that yields the half maximum desensitization rate, and *n* is the Hill coefficient [15]. It was assumed that calcineurin increased *d*_2_ by increasing its [GABA] dependency through a reduction of *k*_h_, and the *d*_2_ and *k*_off_ were changed between the simulated wild-type and PRIP-DKO pyramidal cells. These changes were comparable to those caused by the activation of calcineurin reported previously [4,13].

In this simulation, the onset and offset of the 2 s puff application of GABA were assumed to be attenuated with a time constant raging between 0.1 and 0.3 s. In the simulated wild-type pyramidal cell, GABA_A_R-mediated currents were induced without a hump-like tail-current in response to 2 s GABA puff at 50 µM [15]. In contrast, in the simulated PRIP-DKO pyramidal cell, GABA_A_R-mediated currents displayed a prominent desensitization and were followed by a prominent hump-like tail-current [15]. Thus, a slowdown of *k*_off_ and an acceleration of *d*_2_ resulted in a generation of a hump-like tail-current. Following a sharp decrease in [GABA] at the offset of GABA puff, a sharp decrease in *d*_2_ to a level smaller than the fast de-desensitization (i.e., resensitization) rate constant (*r*_2_) occurred to subsequently induce a hump-like tail-current. Indeed, decreases in the decay time constant at the offset of GABA puff pulse from 0.3 to 0.1 sec decreased the half-duration of the hump-like tail-current, leaving its amplitude almost unchanged [15]. Only PRIP-DKO pyramidal cells, but not wild-type pyramidal cells, displayed hump-like tail-currents in response to the same GABA puff that may have decayed slowly. These observations clearly indicate that the generation of the hump-like tail-current reflects kinetic differences between GABA_A_R-mediated currents in wild-type and PRIP-DKO pyramidal cells. Taken together, it can be concluded that a higher calcineurin activity in PRIP-DKO layer III pyramidal cells might have caused a slowdown of *k*_off_ and an acceleration of *d*_2_ through the modulation of its GABA concentration dependency, leading to a generation of hump-like tail-currents in PRIP-DKO pyramidal cells.

Because there were no significant differences in the single-channel current and the number of GABA_A_Rs between eIPSCs in PRIP-DKO and wild-type pyramidal cells [35], it can be investigated whether the increase in *d*_2_ and the decrease in *k*_off_ can also lead to the prolongation of eIPSCs. Simulated IPSCs in PRIP-DKO and the wild-type pyramidal cells that have half-durations similar to those obtained in the real experiments [35] revealed that a prolongation of eIPSCs/eIPSPs in PRIP-DKO pyramidal cells results from resensitization of GABA_A_R-mediated currents, which is brought about by an acceleration of *d*_2_ through the modulation of its [GABA] dependency together with a slowdown of *k*_off_. The finding of a negative skewness coefficient in PRIP-DKO eIPSCs obtained by the nonstationary variance analysis [35] is consistent with the occurrence of de-desensitization (resensitization) of GABA_A_R-mediated currents during the decay phase of PRIP-DKO eIPSCs.

**Figure 4:**
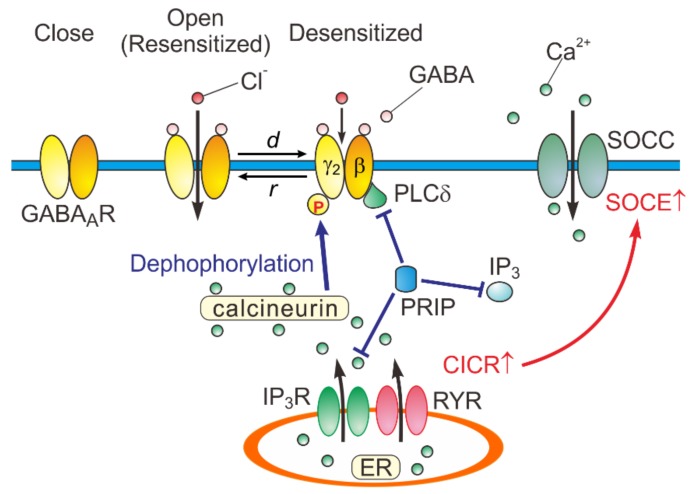
Close, open (resensitized), and desensitized states of GABA_A_Rs. When GABA binds to GABA_A_Rs, the receptors open the pore and consequently increase the permeability of the ion pore to Cl^-^. In response to a prolonged application of GABA, GABA_A_Rs are desensitized (*d*) by increased calcineurin activity due to potentiated Ca^2+^-induced Ca^2+^ release (CICR) followed by store-operated Ca^2+^ entry (SOCE) [15]. GABA_A_Rs are resensitized through de-desensitization (*r*) at the offset of the GABA puff. PRIP outcompetes the PLCδ in binding to GABA_A_R β subunits [40]. *d*: desensitization, *r*: resensitization, RYR: ryanodine receptor, SOCC: store-operated Ca^2+^ channel, IP_3_R: inositol trisphosphate receptor.

Based on the experimental and simulation studies, the regulatory mechanisms of GABA_A_Rs are schematically depicted (Figure 4).

## 6. Physiological Significance of Desensitization and Resensitization of GABA_A_R-Mediated Currents

A single whisker deflection elicits an excitation in a subset of layer IV neurons within a single barrel-related column [41], which subsequently causes an excitation in layer II/III in the same column and then spreads horizontally into neighboring columns [42,43]. The spatio-temporal profile of the excitation spread in layer II/III evoked by stimulation of layer IV was narrower and faster in the barrel cortex of the PRIP-DKO mice compared to the wild-type mice [35].

Such a horizontal excitation spread in layer II/III seems to be strictly controlled by GABA_A_R-mediated lateral inhibition [42,44,45]. Indeed, bicuculline application abolished such a difference in the spatio-temporal profile of the excitation spread in layer II/III between the two genotypes [35]. It is reported that the PRIP-DKO mice exhibited a greater decrease in performance in the rotarod test [36], which is commonly used to assess the sensorimotor integration [46]. Then, the enhanced phasic inhibition caused by the PRIP-1/2 deletion would suppress the inter-columnar integration in the barrel cortex, consequently decreasing spatial recognition. Further studies are required to clarify the roles of PRIP-1/2 in sensorimotor processing in the barrel cortex.

## 7. Clinical Significance of Desensitization and Resensitization of GABA_A_R-Mediated Currents

Central nervous system depressants slow brain activity, making them useful for treating anxiety, panic, and sleep disorders. Alcohol and benzodiazepine are useful to mitigate anxiety through enhancing GABA_A_R-mediated inhibition. However, alcohol and benzodiazepine are known as abused drugs. Alcohol or benzodiazepine withdrawal syndrome appears following a reduction in alcohol or benzodiazepine use after a period of excessive use [47,48,49,50]. The alcohol or benzodiazepine withdrawal symptoms typically include anxiety, sweating, hand tremor, and sleep disturbance. The underlying mechanisms involve neuronal adaptations, which are revealed as decreased GABAergic responses [51] and enhancement of NMDA responses [52,53,54,55]. Although the exact mechanism for the reduced responsiveness of GABA_A_Rs remains uncertain, changes in surface GABA_A_R protein level and subunit composition, changes in turnover, recycling, and production rates, degree of phosphorylation, and decreased coupling mechanisms between GABA and alcohol/benzodiazepine sites are thought to be involved in the reduced responsiveness [56,57,58,59]. It has recently been demonstrated that the benzodiazepine diazepam caused downregulation of GABAergic inhibition through the phospholipase C (PLCδ)/Ca^2+^/calcineurin signaling pathway [40]. The study showed that overexpression of PRIP-1 suppressed diazepam-dependent activation of PLCδ and diazepam-dependent downregulation of GABA_A_Rs in HEK293 cells [40], indicating that PRIP-1 acts as an inhibitor by outcompeting the PLCδ binding to GABA_A_Rs. Because intracellular Ca^2+^ and calcineurin activity are increased in PRIP-DKO mice [15], these findings suggest that the diazepam-induced long-term downregulation of GABAergic inhibition is mediated by the PLCδ/Ca^2+^/calcineurin signaling pathway. Nevertheless, it is also true that calcineurin causes resensitization of GABA_A_R-mediated currents by facilitating their desensitization [4,15]. Given the apparently contradictory behaviors of GABA_A_R-mediated currents by calcineurin activation, the two different behaviors of GABA_A_R-mediated currents may depend on whether calcineurin activation occurs before or after activation of GABA_A_Rs.

As for the treatment of benzodiazepine/alcohol withdrawal syndrome, propofol and barbiturate which enhance GABA_A_R-mediated inhibition are useful. Indeed, it was demonstrated that propofol and barbiturates (pentobarbital and phenobarbital) were effective for the treatment of alcohol withdrawal syndrome [30,32] and barbiturate (pentobarbital) was effective for the treatment of benzodiazepine withdrawal syndrome [60]. However, it remains unclear how propofol and barbiturate ameliorate reduced GABA responsiveness in patients with benzodiazepine/alcohol withdrawal syndrome. Although the concentrations of propofol and barbiturates that generated the hump-like current are very high [19,21,22] compared to the dose used for treatment of the withdrawal syndrome [30,32], the generation of hump-like GABA_A_R currents itself may suggest the occurrence of resensitization of GABA_A_R-mediated currents. Indeed, the desensitization and deactivation of GABA_A_R-mediated currents are facilitated and slowed, respectively, by propofol/barbiturate at much lower concentrations [19,22]. Then, propofol and barbiturate may improve the reduced GABA responsiveness through the resensitization of GABA_A_R-mediated currents. Therefore, the regulatory mechanisms of desensitization/resensitization of GABA_A_R-mediated currents are important to better understand benzodiazepine/alcohol withdrawal syndrome and to develop the treatment method.

## Figures and Tables

**Table 1:** Kinases and molecules involved in desensitization and slowdown of deactivation of GABA_A_R-mediated currents.

Kinases/ Molecules	Neuron/Recombinant GABA_A_Rs	Effects	References
PKA	Chick cortical neurons	increases desensitization	[7]
	Rat sympathetic ganglion neurons	decreases peak amplitude and increases fast desensitization	[8]
	Rat cerebellar granule cells	decreases fast desensitization	[9]
	α1β1γ2S, α1β3γ2LS	increases desensitization and slows deactivation	[10]
PKC	α1β1	decreases fast desensitization	[11]
PKG	Rat cerebellar granule cells	decreases fast desensitization	[9]
CaMKII	Rat spinal dorsal horn neurons	decreases desensitization	[12]
Calcineurin	Rat hippocampal neurons	increases desensitization and slows deactivation	[4]
PRIP	Mouse cortical pyramidal neurons	PRIP deletion increases desensitization and generates hump-like currents through increased calcineurin activity	[15]
GABARAP	α1β2γ2L	promotes clustering of GABA_A_Rs, facilitates deactivation, and slows desensitization	[18]

**Table 2:** Drugs that modulate GABA responses and directly activate GABA_A_Rs at higher concentrations.

Drugs	Neurons/ Recombinant GABA_A_Rs	Effects	Refs.
Anesthetics			
Propofol	Mouse hippocampal neurons	slows deactivation and increases apparent desensitization of GABA responses at low concentrations and directly elicits after-responses upon washout at high concentrations	[19]
Etomidate	Rat spinal dorsal horn neurons	slows deactivation of GABA responses at low concentrations while directly eliciting tail currents upon washout at high concentrations	[20]
Barbiturate			
Pentobarbital	Frog sensory neurons	slows deactivation and increases apparent desensitization of GABA responses at low concentrations and directly elicits hump currents upon washout at high concentrations	[21,22]
	Rat hippocampal neurons	slows deactivation and increases apparent desensitization of GABA responses at low concentrations and directly elicits rebound currents upon washout at high concentrations	[23]
	α1β2γ2L	directly elicits tail currents upon washout at high concentrations	[24,26]
	α1β3γ2L	slows deactivation and increases apparent desensitization of GABA responses at low concentrations and directly elicits rebound currents upon washout at high concentrations	[25]
	α1β2γ2S, α6β2γ2S	directly elicits hump currents upon washout at high concentrations	[27]
	β3	increases apparent desensitization of GABA responses and directly elicits rebound currents upon washout at high concentrations	[28]
	α1β3γ2L	directly elicits tail currents upon washout at high concentrations	[29]
Phenobarbital	Rat hippocampal neurons	slows deactivation and increases apparent desensitization of GABA responses at low concentrations and directly elicits rebound currents upon washout at high concentrations	[23]

## Abbreviations

CaMKII	Ca^2+^/calmodulin-dependent protein kinase II
CICR	Ca^2+^-induced Ca^2+^ release
DKO	double-knockout
GABA_A_R	GABA_A_ receptor
GABARAP	GABA_A_R-associated protein
IPSC	inhibitory postsynaptic current
IPSP	inhibitory postsynaptic potential
NMDA	N-methyl-D-aspartate
PLC	phospholipase C
PRIP	phospholipase C-related catalytically inactive protein
RYR	ryanodine receptor
SOCC	store-operated Ca^2+^ channel
SOCE	store-operated Ca^2+^ entry
